# Generation of Otic Sensory Neurons from Mouse Embryonic Stem Cells in 3D Culture

**DOI:** 10.3389/fncel.2017.00409

**Published:** 2017-12-19

**Authors:** Michael Perny, Ching-Chia Ting, Sonja Kleinlogel, Pascal Senn, Marta Roccio

**Affiliations:** ^1^Neuroinfection Laboratory, Institute for Infectious Diseases, University of Bern, Bern, Switzerland; ^2^Laboratory of Inner Ear Research, Department for BioMedical Research, University of Bern, Bern, Switzerland; ^3^Department of Otorhinolaryngology, Head and Neck Surgery, Inselspital, University of Bern, Bern, Switzerland; ^4^Cluster for Regenerative Neuroscience, Department of Biomedical Research, University of Bern, Bern, Switzerland; ^5^Institute for Physiology, University of Bern, Bern, Switzerland; ^6^Department of Otorhinolaryngology, Head and Neck Surgery, University Hospital Geneva (HUG), Geneva, Switzerland

**Keywords:** spiral ganglion neurons, organoids, *in vitro* mESC differentiation, otic development, 3D culture

## Abstract

The peripheral hearing process taking place in the cochlea mainly depends on two distinct sensory cell types: the mechanosensitive hair cells and the spiral ganglion neurons (SGNs). The first respond to the mechanical stimulation exerted by sound pressure waves on their hair bundles by releasing neurotransmitters and thereby activating the latter. Loss of these sensorineural cells is associated with permanent hearing loss. Stem cell-based approaches aiming at cell replacement or *in vitro* drug testing to identify potential ototoxic, otoprotective, or regenerative compounds have lately gained attention as putative therapeutic strategies for hearing loss. Nevertheless, they rely on efficient and reliable protocols for the *in vitro* generation of cochlear sensory cells for their implementation. To this end, we have developed a differentiation protocol based on organoid culture systems, which mimics the most important steps of *in vivo* otic development, robustly guiding mouse embryonic stem cells (mESCs) toward otic sensory neurons (OSNs). The stepwise differentiation of mESCs toward ectoderm was initiated using a quick aggregation method in presence of Matrigel in serum-free conditions. Non-neural ectoderm was induced via activation of bone morphogenetic protein (BMP) signaling and concomitant inhibition of transforming growth factor beta (TGFβ) signaling to prevent mesendoderm induction. Preplacodal and otic placode ectoderm was further induced by inhibition of BMP signaling and addition of fibroblast growth factor 2 (FGF2). Delamination and differentiation of SGNs was initiated by plating of the organoids on a 2D Matrigel-coated substrate. Supplementation with brain-derived neurotrophic factor (BDNF) and neurotrophin-3 (NT-3) was used for further maturation until 15 days of *in vitro* differentiation. A large population of neurons with a clear bipolar morphology and functional excitability was derived from these cultures. Immunostaining and gene expression analysis performed at different time points confirmed the transition trough the otic lineage and final expression of the key OSN markers. Moreover, the stem cell-derived OSNs exhibited functional electrophysiological properties of native SGNs. Our established *in vitro* model of OSNs development can be used for basic developmental studies, for drug screening or for the exploration of their regenerative potential.

## Introduction

Spiral ganglion neurons (SGNs) within the cochlea play a central role for sound perception, providing afferent neurotransmission to the central auditory system. Upon activation, they encode frequency, duration, and intensity of all sounds and relay this information to the brain stem and further to higher auditory centers (Appler and Goodrich, 2011; Dabdoub et al., 2016). SGNs, much like cochlear hair cells, are sensitive to insults, including noise overexposure, and do not regenerate after cell death. Therefore, their loss leads to permanent hearing deficit (Lang, 2016).

The loss of hearing due to death or malfunctioning of hair cells can be successfully restored by a cochlear implant (CI), an electrode-array-based neuroprosthesis, which directly stimulates SGNs (Clopton et al., 1980; O’Donoghue, 2013; Boulet et al., 2016). However, a sufficient number of SGNs is required for their functioning. Retrospective studies revealed a correlation between the SGN density and the success of the implant (Blamey, 1997; Incesulu and Nadol, 1998; Fayad and Linthicum, 2006).

Strategies aiming at regenerating or replacing lost SGNs could complement and increase the success of these devices. Therefore, generating mature SGNs *in vitro* that could be used for regenerative therapies has been a long sought goal (Martinez-Monedero and Edge, 2007; Geleoc and Holt, 2014; Muller and Barr-Gillespie, 2015). Pre-clinical strategies to use cell-therapy for SGN replenishment consist of two distinct approaches, namely, *in vivo* activation of local progenitors (either chemically or genetically) or cell transplantations. Somatic SGN progenitors or SGNs derived from pluripotent cells appear to be the most suitable cell sources for these approaches.

A tissue resident source of progenitors seems to be represented by Schwann cells in the ganglion. These cells have been shown to proliferate after chemical ablation of SGNs with Ouabain (Lang et al., 2011). However, they did not differentiate to neurons under these conditions. *In vitro* lineage tracing has demonstrated, however, that these cells are capable to differentiate into neurons and other glial cells (McLean et al., 2016) and seem to represent the population of cells that can be expanded as neurospheres *in vitro* upon isolation from young postnatal animals (Oshima et al., 2007a,b; Lang et al., 2015). Identification of signaling pathways enhancing their neuronal differentiation could lead to a drug-based therapy, thereby promoting their proliferation or neuronal differentiation *in situ* (Song et al., 2017). Alternatively, *in vivo* reprograming through gene therapy could lead to their neuronal differentiation.

*Ex vivo* cultured/expanded neurosphere-forming cells from the spiral ganglion have been shown to differentiate *in vitro* to neurons and re-innervate a denervated organ of Corti explants (Martinez-Monedero et al., 2008) and could also be suitable for *in vivo* transplantations (Martinez-Monedero et al., 2007). However, the clinical setting of such a therapy would likely rely on the donation of rare human fetal material.

*In vitro*-generated SGNs from pluripotent stem cells represent an interesting alternative to somatic progenitors. They could be used for cell transplantation/replacement strategies to replenish lost neurons (Chen et al., 2012). Alternatively, they could be exploited *ex vivo* for drug-testing (Whitlon et al., 2015) or for the optimization of CI stimulation protocols, by studying the optimal electrical stimulation parameters (Hahnewald et al., 2016).

The challenge in generating otic SGNs from pluripotent cells consists in finding the suitable culture conditions to guide cells through the stages of *in vivo* organ development and to prove the otic nature of the generated neurons. Due to the lack of specific unambiguous markers in the mature stage, it is of critical importance to document and verify the *in vitro* differentiation steps through which these neurons have transited in order to verify their lineage.

The neurosensory cells of the inner ear are derived from the otic vesicle (Magarinos et al., 2012; Delacroix and Malgrange, 2015; Goodrich, 2016). The otic vesicle derives from non-neural ectoderm (NNE), which is induced shortly after gastrulation from the ectoderm layer by a lateral-to-medial gradient of bone morphogenetic protein (BMP) signaling (Wilson and Hemmati-Brivanlou, 1995; Barth et al., 1999). NNE is then specified to become pre-placodal ectoderm (PPE) at the border between the developing epidermis and neural ectoderm (Kwon and Riley, 2009; Kwon et al., 2010; Steventon et al., 2014). Down-regulation of BMP signaling and activation of fibroblast growth factor (FGF) signaling have been shown to be important for PPE development (Glavic et al., 2004; Martin and Groves, 2006). From the PPE, all cranial placodes are generated, including the otic placode (Streit, 2004). FGF signaling, generated by the hindbrain and underlying mesenchyme, is essential for this induction step. Coordinated and redundant effect of different FGF family members (FGF3, FGF8, and FGF10) contributes to this process (Alvarez et al., 2003; Pauley et al., 2003; Wright, 2003; Zelarayan et al., 2007). Subsequently, the otic placode invaginates from the surface ectoderm and generates the otic vesicle, which harbors the progenitors of cochlear/vestibular hair cells but also the otic sensory neurons (OSNs). Neurogenesis of SGNs starts at around E9.0 in the proneurosensory domain and is followed by the delamination and epithelial-to-mesenchymal transition of a population of cells from the otic vesicle (Yang et al., 2011; Magarinos et al., 2012). The upregulation of Neurogenin1 (Ma et al., 2000) and subsequently NeuroD1 (Kim et al., 2001; Evsen et al., 2013) in the proneurosensory domain is initiating the specification of a neural fate within the Sox2 expressing domain in the otocyst. This pool of cells will give rise to the SGNs and vestibular ganglion (VG) neurons during later development (reviewed in Appler and Goodrich, 2011).

Previous studies have shown the generation of otic-like neurons from multi- and/or pluripotent cells by the manipulation of the FGF-, BMP, and Sonic hedgehog-signaling pathways and the supplementation of neurotrophic factors [i.e., brain-derived neurotrophic factor (BDNF) and NT3] (Kondo et al., 2005, 2011; Shi et al., 2007; Chen et al., 2012). However, the focus was not set on the detailed step-by-step differentiation through the otic lineage during the proposed protocols.

Using 3D culture methods, Koehler and colleagues recently showed that mouse and human pluripotent stem cells could be induced by growth factors and small molecule inhibitors to recapitulate the developmental process *in vitro*, leading to differentiation of sensory hair cells in 3D organoids. Accompanying hair cells, a number of neurons were generated within these organoids, making synaptic contacts with the hair cells. Some of these neurons expressed calretinin, Brn3a, and Islet1. However, the origin, nature, and functionality of these neurons were not further investigated (Koehler et al., 2013; Koehler and Hashino, 2014). Their protocol relied on initial transient induction of definitive ectoderm (DE), using previously described methods (Eiraku et al., 2011), and subsequent NNE induction by providing BMP4 to the culture. Induction of PPE *in vitro* was achieved by inhibition of BMP signaling and activation of FGF signaling.

Extending on these findings and protocols, we have further assessed the possibility of deriving otic neurons *in vitro*. We show here the stepwise generation of high number of otic bipolar neurons expressing key gene/protein markers and electrophysiological properties of native SGN.

## Materials and Methods

### Mouse Embryonic Stem Cell (mESC) Culture

Mouse embryonic stem (E14) cells were cultured in LIF-2i medium on 0.1% gelatin-coated culture plates. Equal volumes of DMEM/F12 (Invitrogen, United states) and Neurobasal medium (Invitrogen, United States) supplemented with N2/B27, 1 mM Glutamax, 1,000 U ml^-1^ leukemia inhibitory factor (LIF; Merck Millipore, catalog number: ESG1107), 1 μM PD03259010, and 3 μM of CHIR99021.

Upon thawing, cells were initially plated in a mix of 75% LIF-2i medium and 25% “Serum-LIF” medium. The latter containing DMEM, 15% embryonic stem cell grade fetal bovine serum (FBS; Thermo Fisher, Cat. No. 16141061), 1 mM of penicillin/streptomycin, 1 mM of non-essential amino acid, 0.1 mM of 2-mercaptoethanol, and 1,000 U ml^-1^ LIF. mESCs were incubated at 37°C with 5% CO_2_ overnight, before medium was changed to 100% LIF-2i on the following day. When cell density reached 80% confluence, cells were detached and passaged with 0.25% trypsin-EDTA for 1–2 min at 37°C.

### Differentiation into Otic Sensory Neurons (OSNs)

Serum-free embryoid body quick (sfEBq) aggregation and initial differentiation were performed as previously described (Eiraku et al., 2011; Koehler and Hashino, 2014). In brief, 3,000 mESCs per well were aggregated on day 0 at the bottom of U-shaped low adhesion 96-well plates in ectoderm differentiation medium [G-MEM with 1.5% knockout serum replacement (KSR), 0.1 mM non-essential amino acids, 1 mM sodium pyruvate, 1 mM penicillin/streptomycin, and 1 mM 2-mercaptoethanol]. The following day (day 1), half of the medium was exchanged for differentiation medium containing 2% Matrigel (v/v final concentration). On day 3, recombinant BMP-4 (R&D) 10 ng/ml together with 1 μM of transforming growth factor beta (TGF-β) inhibitor SB 43-1542 (Stemgent) was supplemented to the medium. At day 4½to day 5, 25 ng/ml FGF-2 (Peprotech), and 1 μM BMP inhibitor LDN-193989 (Stemgent) were added to the culture.

Organoids were then plated on Matrigel-coated well plates or glass slides. Matrigel was diluted for coating 1:4 or 1:10 depending on the experiment. On day 8, after assessing attachment of the organoids to the culture plate, medium was changed to OSN medium, containing: DMEM/F12, B27, N2, NT3 (5 ng/ml) (Peprotech Cat. No. 450-03), and BDNF (5 ng/ml) (R&D Cat 248-BD). Medium was changed every other day until termination of the experiment.

### RNA Isolation

RNA was isolated using Trizol^®^. Thirty organoids plated in a single well of a six-well plate coated with Matrigel^®^ were lysed in 1 ml of Trizol^®^ after culture for 8, 12, or 15 days. For day 5 organoids, *n* = 30 were harvested prior to plating. Lysis was performed by repetitive pipetting. Chloroform separation was performed according to manufacturer’s instruction. After phase separation step, the RNA containing supernatant was moved into a new eppendorf tube, and 1 volume of 70% ethanol was added, and mixed gently until homogenization. The mixture was then loaded onto the RNeasy spin column from RNeasy^®^ Plus Mini Kit (Qiagen), and proceeded as manufacturer’s instructions. RNA was eluted in a volume of 20 μl of RNAse-free water and quantified using nanodrop (Thermo Fisher Scientific).

### cDNA Synthesis

cDNA was synthesized using Bio-Rad iScript cDNA Synthesis Kit according to manufacturer’s instruction starting from 1 μg RNA. After synthesis, cDNA was diluted 1:10 in RNAse-free water.

### qPCR

SYBR^®^ Select Master Mix from Applied Biosystems was used for qPCR. The PCR was run on a TaqManViia^TM^ 7 instrument. Primers were designed using primer BLAST and selected to span exon–intron boundaries. Data are normalized to Beta actin expression, run for each plate for all samples, and expressed as fold-change to mESC in the undifferentiated state (d0) using the formula 2^-ΔΔ*C*_T_^. qPCR data are the average of two to five independent experiments as indicated in the figure legend.

### Immunofluorescence

Samples were fixed with 4% PFA for 10 min at room temperature, subsequently washed with PBS, permeabilized, and blocked in blocking solution (with 2% BSA, 0.1% Triton-X100 in PBS) for 2 h. Primary antibodies were added at 1:100 dilution [rabbit polyclonal anti-MyoVIIa (Proteus); mouse monoclonal anti-Sox2 (Millipore); rabbit polyclonal anti-Sox2 (Invitrogen); rat anti-E-cadherin (Abcam); mouse anti-GATA3 (Thermo Fisher Scientific); mouse anti-Islet 1 (DSHB, deposited by Jessell T.M.); goat anti-Doublecortin (Santa Cruz Biotechnology); rabbit anti-Pax2 (Thermo Fisher Scientific); rabbit anti-Pax8 (Abcam); mouse anti-Nestin (BD Transduction Laboratories); mouse anti-βIII-Tubulin (R&D); rabbit anti-Peripherin (Millipore); and mouse monoclonal anti-Brn3a (Millipore)], and incubated in blocking solution overnight at 4°C. Samples were then washed three times with PBS, followed by the addition of Alexa Fluor conjugated secondary antibodies (Invitrogen) at 1:500 dilution in blocking buffer for 2 days at room temperature. The images were acquired with a confocal microscope (Zeiss LSM 700) using 10× and 20× air objectives.

### Electrophysiological Characterization of OSNs

The aggregates were plated on day 5 of differentiation on laminin-coated (0.1 mg/ml, Sigma) coverslips for electrophysiological patch-clamp recordings. Matrigel^®^ coating was not compatible for this assay.

Whole-cell patch-clamp recordings from the cell somata were performed at room temperature on an inverted Zeiss Axiovert 35 M microscope using borosilicate glass pipettes (Harvard Apparatus GC150F-10) pulled with a Zeitz DMZ-Universal puller with resistances ranging from 3 to 6 MΩ. The pipette solution contained (in mM): 123 K-gluconate, 7 KCl, 1 MgCl_2_, 5 Na_2_-ATP, 10 EGTA, 10 HEPES; pH 7.35 (KOH), 285–290 mOsm. The bath solutions contained (in mM): 135 NaCl, 5.8 KCl, 0.9 MgCl_2_, 1.3 CaCl_2_, 5.4 D-glucose, 10 HEPES, 0.7 NaH_2_PO_4_, and 2 Na-pyruvate (pH 7.35). Liquid junction potentials were corrected for all experiments. Signals were amplified with an Axopatch 200B Amplifier, low pass filtered at 5 kHz, and digitized at 10 kHz with an Axon Digidata 1440A. Data acquisition and analysis were performed using pClamp software (Molecular Devices, Biberach, Germany). Whole-cell current-clamp experiments were performed with 0 pA holding currents and spiking was initiated by current steps from +5 to +65 pA in 10-pA increments.

### Statistical Analysis

Gene expression analysis at the different time points was analyzed for statistical significance using one-way ANOVA. Each time point represents the mean of three to five independent experiments expressed as fold change vs. ESC (d0). The means of each group were compared to the mean of all other groups and Tukey’s test for multiple comparison correction was applied (black lines). Additionally, uncorrected Fisher’s LSD test is indicated by gray lines. Statistical analysis was performed using Graph-Pad Prism 7.

## Results

### Sensory Neurons “Delaminate” from 3D Inner Ear Organoids

Previous reports had elegantly demonstrated the *in vitro* generation of human and mouse otic vesicle-like structures and subsequent differentiation into sensory hair cells from pluripotent stem cells using 3D culture methods (Koehler et al., 2013, 2017; Koehler and Hashino, 2014). Here, we assessed the possibility to induce an *in vitro* “delamination” of neuroblasts from otic placode structures generated during this differentiation approach.

**FIGURE 1:**
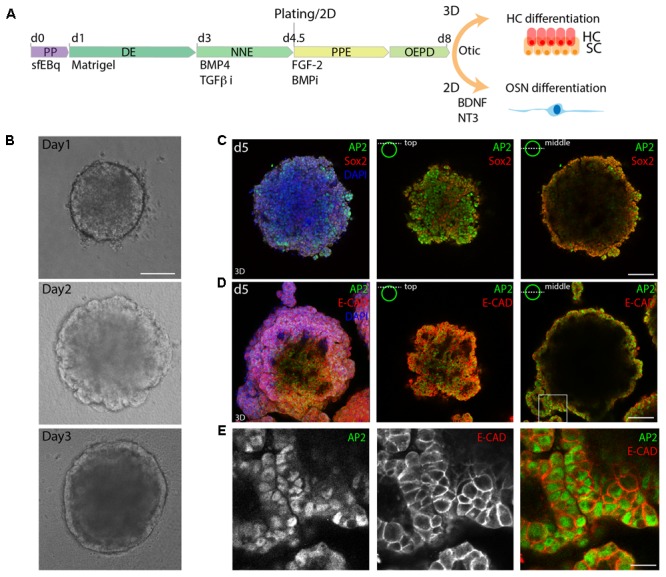
3D *in vitro* formation of non-neural ectoderm. **(A)** Schematic of the differentiation protocol. PP, pluripotent; DE, definitive ectoderm NNE, non-neural ectoderm; PPE, pre-placodal ectoderm; OEPD, otic epibranchial placode domain; HC, hair cells; OSN, otic sensory neurons (OSNs). **(B)** Morphological changes during the first 3 days of differentiation are visualized by brightfield microscopy. Scale bar 100 μm. **(C)** Immunostaining of day 5 (d5) organoids for Sox2 and AP2. **(D)** Immunostaining of day 5 organoids for AP2 and Ecad. 3D maximum projection (3D) and single stack (top and middle) are shown for the acquired confocal stacks. Scale bar 100 μm. **(E)** Magnified view of the boxed area in **D**, showing the formed AP2^+^, Ecad^+^ outer epithelium. Scale bar 20 μm.

A schematic of the protocol is shown in **Figure 1A**. The differentiation protocol was started by the induction of DE by using a serum-free quick aggregation protocol (sfEBq) in presence of Matrigel and KSR. On day 3, the aggregates were further guided to differentiate to NNE by activating BMP4 signaling. Simultaneously, the TGF-β inhibitor SB43-1542 (1 μM) was applied to inhibit mesoderm induction. PPE, followed by otic epibranchial placode domain (OEPD), was induced between day 5 and day 8 with a concomitant addition of recombinant FGF-2 and inhibition of BMP signaling, using LDN193989 (1 μM). This last step was performed in a 3D/2D setup by plating the aggregates on Matrigel-coated substrates, in contrast to the previously described protocol for hair cell generation. Delamination and maturation of OSNs were initiated on day 8 with the supplementation of BDNF and NT-3 to the medium until day 12 or day 15 of differentiation.

Similarly to previous studies, we morphologically identified a DE layer on the outside of the organoid on day 3 of sfEBq culture (**Figure 1B**). Subsequently, the expression of the NNE marker activator protein 2 (AP-2) in the E-cadherin positive outer epithelium was observed at day 5 (**Figures 1C–E**), indicating the formation of NNE.

**FIGURE 2:**
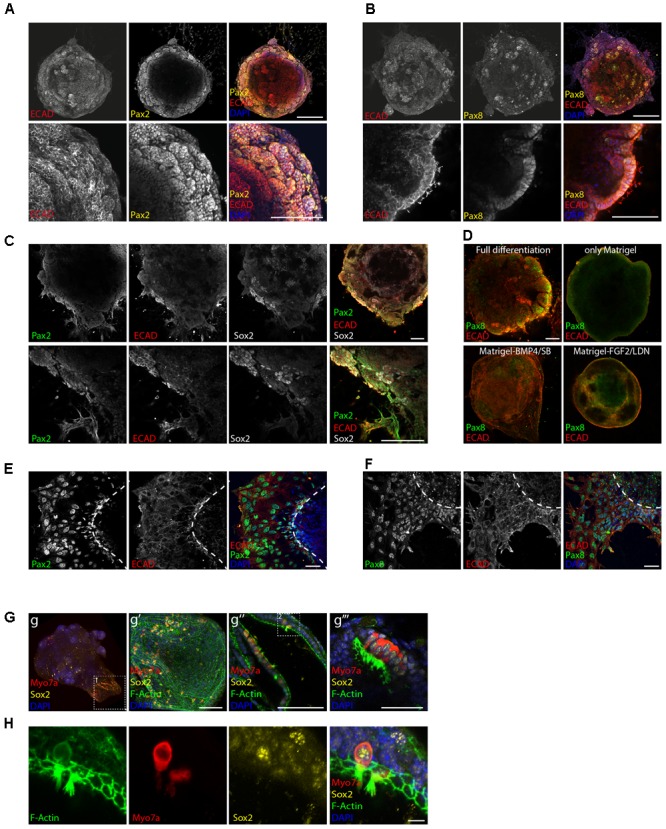
Induction of otic placode fate. Expression of the otic placode markers Pax2 **(A)** and Pax8 **(B)** together with Ecad on day 8 of differentiation. Scale bars 100 μm. **(C)** Co-expression of Pax2, Ecad, and Sox2 on day 8 of differentiation. Scale bars 100 μm. **(D)** Organoids cultured in full or “partial differentiation media” (Only Matrigel, Matrigel-BMP4/SB, or Matrigel-FGF2/LDN) are immunostained for Ecad and Pax8 at day 8 of differentiation. Scale bars 100 μm. **(E)** Immunostaining at day 8 of differentiation for the otic markers Pax2 and Ecad and **(F)** Pax8 and Ecad after organoid attachment and delamination. Scale bars 50 μm. **(G)** Myosin7a^+^, Sox2^+^ hair cells were obtained 20 days after differentiation. Confocal images of the whole organoid and selected areas expressing hair cells and supporting cells are shown. Boxed area 1 (in g) enlarged in g′ panel. 3D projection is shown. Scale bar 100 μm. Hair cells containing regions from two organoids are shown. g″ (scale bars 100 μm) g″′ (scale bar 50 μm). Boxed area 2 (in g″) enlarged in **H**. Single channels for a selected stack are shown. Scale bars 10 μm.

In agreement with the previously published literature, E-cadherin positive otic vesicle-like structures expressing the otic markers Pax2 and Pax8 (Bouchard et al., 2010; Koehler et al., 2013) were formed at the periphery of the aggregates on day 8 of differentiation (**Figures 2A,B**). Sox2 positive patches were instead identified as expected both in the core of the organoid, marking remaining pluripotent cells, as well as in the periphery, where partial localization with Pax2 was observed (**Figure 2C**). This was in agreement with the role of Sox2 in neurosensory cell development (Dvorakova et al., 2016).

Differentiation protocols where we omitted one of the above-mentioned guidance steps (only Matrigel, Matrigel-BMP4/SB, Matrigel-FGF2/LDN) failed to generate otic tissue as shown by the absence of otic vesicle-like structures (**Figure 2D**) and by gene expression analysis (**Supplementary Figures S1A,B**).

Certain parts of the aggregates were already strongly adherent to the substrate with cells attaching to the surface and migrating out at day 8. This was accompanied by a disorganization of the epithelial layer. Despite the strong structural changes, the adherent cells remained immunopositive for the otic markers Pax2 and Pax8 at this time point (**Figures 2E,F**).

Maintaining the aggregates in previously developed media for hair cell differentiation/maturation (Koehler et al., 2013) and in suspension culture, indeed lead to generation of hair cells, again supporting the hypothesis that we had steered tissue development *in vitro* toward the otic lineage (**Figures 2G,H**). Hair cells appeared at day 20 of differentiation expressing the hair cell marker Myosin7a and F-actin^+^ hair bundles. The Myosin7a^+^ cells were embedded in Sox2^+^ supporting cells and still co-expressed Sox2, indicative of their immature stage (Dabdoub et al., 2008).

### Otic Sensory Neuron Differentiation and Maturation

**FIGURE 3:**
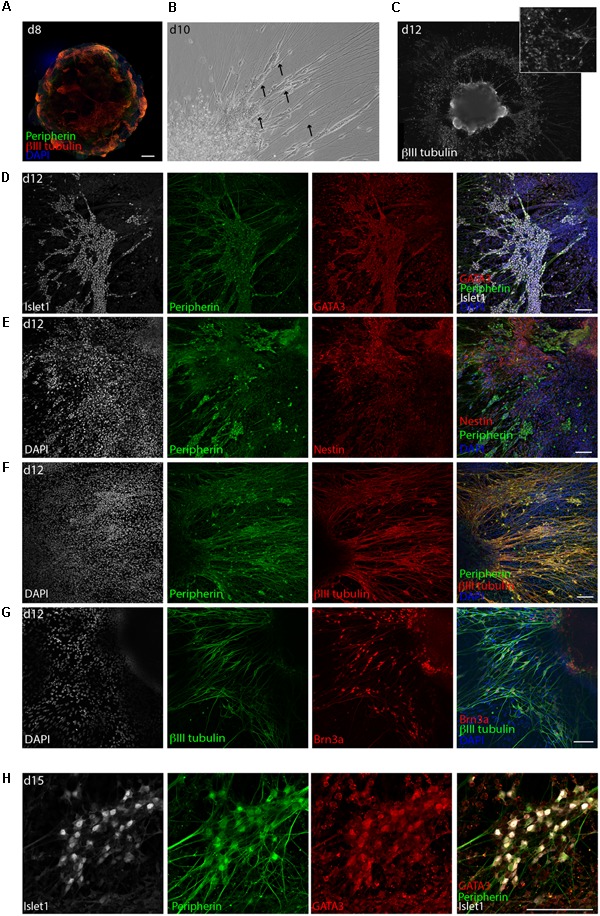
Delamination of otic neuroblasts and differentiation into sensory neurons. **(A)** Representative example of day 8 organoid immunostained for βIII-tubulin and peripherin. Scale bar 100 μm. **(B)** Brightfield image of a day 10 organoids showing neuronal delamination. Arrows point at groups of neuronal somas migrating from the organoids in clusters. **(C)** Immunostaining for βIII-tubulin of a day 12 organoid. Formation of ganglion-like structure with neuronal somas migrating outside of the organoid is illustrated. **(D)** Islet1, GATA3, and peripherin expression at day 12 of differentiation. Scale bar 100 μm. **(E)** Peripherin and nestin expression at day 12 of differentiation. Scale bar 100 μm. **(F)** Peripherin and βIII-tubulin expression at day 12. Scale bar 100 μm. **(G)** βIII-tubulin and Brn3a expression at day 12. Scale bar 100 μm. **(H)** Islet1, GATA3, and peripherin expression at day 15 of differentiation. Scale bar 100 μm.

Already on day 8, the surface of the aggregates was containing high numbers of cells expressing the neuronal marker βIII-tubulin together with the intermediate filament peripherin (**Figure 3A**). BDNF and NT3 are known neurotrophins (NT) involved in SGN maturation and migration during cochlear development (Fritzsch et al., 2004). Extensive neuronal outgrowth was observed upon supplementation of the medium with both factors starting on day 8 to induce neuronal differentiation. This started already on day 9/10 (**Figure 3B**). Four days later (day 12), the generated βIII-tubulin^+^ neurons displayed a bipolar morphology. The somas of these neurons migrated out of the core aggregate, forming multiple ganglion-like structures (**Figure 3C** and **Supplementary Figure S1C**).

The derived neurons displayed robust co-expression of the transcription factors GATA3 and Islet1 (**Figure 3D**). Furthermore, the neurons were highly positive for peripherin and Brn3a, which is a marker for peripheral sensory neurons, and were surrounded by Nestin^+^ cells (**Figures 3E–G**). At later time points, namely day 15, βIII-tubulin^+^, peripherin+ neurons were still expressing GATA3 and Islet1 (**Figure 3H**) and grew neurites for several millimeter in culture (not shown). However, at this time the culture started to display signs of cell death probably due to overgrowth.

**FIGURE 4:**
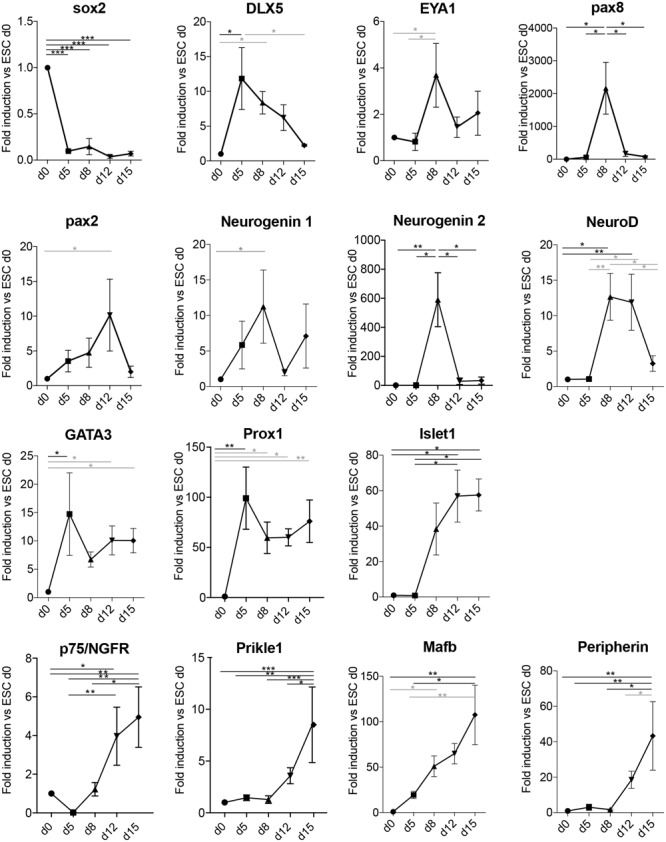
Neuronal differentiation transits through otic developmental steps. Gene expression analysis of the spiral ganglion development-related genes Sox2, Dlx5, Eya1, Pax8, Pax2, Neurogenin 1, Neurogenin 2, NeuroD, GATA3, Prox1, Islet1, p75, Prikle1, Mafb, and Peripherin at the indicated days in culture in differentiation conditions. Values are fold induction versus undifferentiated mESCs (d0, set as 1). For each time point, the average fold induction obtained in three to five independent experiments is shown. d0 (*n* = 5); d5 (*n* = 3); d8 (*n* = 5); d12 (*n* = 4); and d15 (*n* = 3). Error bars are SEM. Black (ANOVA with multiple comparison correction, Tukey’s test); Gray bars (ANOVA w/o multiple comparison correction: Fisher’s LSD test). (^∗^*p* < 0.05, ^∗∗^*p* < 0.01, ^∗∗∗^*p* < 0.005).

In order to assess if the derived neurons had indeed transited through the otic lineage, we assessed a panel of markers known to be important for the early development of the statoacoustic ganglion and later development of SGNs (**Figure 4**). Sox2 expression was strongly downregulated upon differentiation and loss of pluripotency. Peak expression of the PPE and otic placode markers Dlx5, Eya1, and Pax8 were observed in the aggregates between day 5 and day 8 of differentiation. Pax2 was slightly more delayed, peaking at day 12 (Bouchard et al., 2010). Neurogenin 1, as well as Neurogenin 2 expression was peaking on day 8, followed by transient upregulation of NeuroD. Expression of GATA3 and Prox1 (Duncan and Fritzsch, 2013; Nishimura et al., 2017) showed an increased expression from day 5, which was then maintained until the last time point assessed (day 15). Additional SG markers, such as Islet1 (Radde-Gallwitz et al., 2004), the NT receptor p75/NGFR (von Bartheld et al., 1991; Sato et al., 2006), Prikle1 (Yang et al., 2017), and Mafb (Lu et al., 2011) were also up-regulated in a time-dependent manner. The intracellular filament peripherin was one of the latest markers starting to appear at day 12–15 of differentiation, which was concomitant with the morphological appearance of differentiated neuronal cells.

We then assessed neuronal differentiation in partial differentiation protocols with cells exposed either solely to Matrigel (only Matrigel), or only to BMP4 and SB43-1542 (Matrigel-BMP4/SB) or only to FGF2 and LDN193989 (Matrigel-FGF2/LDN) (**Supplementary Figure S1**). Samples where we omitted one or the other step failed to induce key otic markers such as Pax8 and Pax2 at the mRNA levels. The same was observed for other neuronal markers tested such as neurogenin 2, peripherin, and for the SGN markers Mafb and GATA3 (**Supplementary Figure S1B**). While Matrigel-BMP4/SB aggregates developed very few neurons, in agreement with the role of prolonged BMP signaling in inducing epidermis from NNE (Wilson and Hemmati-Brivanlou, 1995), the other two conditions developed neurons that were morphologically distinct compared to the full differentiation protocol. While GATA3 and Islet 1 expression was faintly detectable in some sparse neurons in these conditions and not in clusters, as in the full differentiation protocol, the sensory marker Brn3a could be detected additionally in the Matrigel-only treated organoids (**Supplementary Figures S1C,D**).

### Electrophysiological Analysis of SGN-Like Cells

**FIGURE 5:**
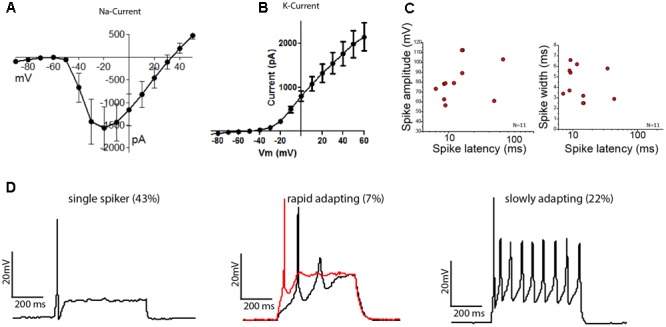
Stem cell-derived otic tissue generates functional OSNs. Current–voltage relationships of sodium **(A)** and potassium **(B)** currents (mean ± SEM, *N* = 14). **(C)** Spike amplitude (mV) (left) and spike width (right) are plotted against the spike latency for *n* = 11 cells. **(D)** Whole-cell current clamp recordings showing different type of firing patterns are illustrated: Single spikers (Left), rapidly adapting [Middle, 11 pA (black trace) and 13 pA (red trace, depolarization block) injected, respectively] and slowly adapting neurons (Right).

Spiral ganglion neuron-like neurons were examined for their electrophysiological properties by whole-cell patch-clamp recordings on day 12 of differentiation (**Figure 5**). Differentiated OSNs had a hyperpolarized, typical neuronal resting membrane potential of -57.3 ± 10.2 mV (mean ± SD, *N* = 16) and expressed voltage-gated Na+ and K+ channels (**Figures 5A,B**). Delayed-rectifier K+ currents (*I*_K_s) activated at a depolarized membrane potential of approximately -45 mV and produced currents of up to 3.5 nA at +40 mV. In contrary, hyperpolarizing voltage steps did not elicit any inward rectifier potassium currents (*I*_K1_) or sustained inward Ca2+ currents (*I*_Ca_, data not shown), as had been described for hair cell-like cells (Chen et al., 2012; Liu et al., 2016).

The cells which possessed K+ and Na+ currents (*N* = 14/16) reliably fired action potentials (AP) when current was injected. We found three types of firing patterns: the majority of neurons fired a single AP when activated (*N* = 6/14), while 22% of neurons showed sustained firing (*N* = 3/14) and 1 out of 14 neurons showed a rapidly adapting firing pattern with depolarization block. The observed patterns align well with the activity patterns reported for acute cultures of mouse SGNs, which depends on their anatomical position along the cochlear axis (Adamson et al., 2002a,b). The average AP-latencies between the onset of the step current injection and the peak of the initial AP were 15.88 ± 3.61 ms (mean ± SEM, *n* = 11). However, the latencies could be grouped into neurons with fast latencies (10.58 ± 2.99 ms, mean ± SD, *n* = 9) and neurons with slower latencies (39.05 ± 6.58 ms, mean ± SD, *n* = 2), in line with the descriptions of SGNs from mouse acute cultures (Adamson et al., 2002b) (**Figure 5C**). The average spike widths (4.35 ± 1.58 ms, mean ± SEM, *n* = 11) and spike amplitudes (81.25 ± 6.13 mV, mean ± SEM, *n* = 11) were also in the range reported for acutely isolated mouse SGNs (Liu and Davis, 2007; Matsuoka et al., 2017). We also found cells that showed immature characteristics despite a neuronal morphology, but these were only 2 of the tested 14 cells.

In agreement with the visual appearance of some dead cells on day 15, electrophysiological recordings also showed a reduced membrane potential and less responsive cells (data not shown).

## Discussion

The ability to generate SGNs from stem cells is required to realize clinical cell-replacement therapies for peripheral hearing loss. We have developed a stepwise protocol, imitating specific development steps, to reliably and reproducibly derive SGN-like cells from mESCs. We showed that the generated cells express appropriate markers of cells transiting through otic development and finally display neuronal morphology and functional properties of OSNs.

The protocol we present here builds on previously well-characterized guidance steps through growth factor and small molecule inhibitor application to re-capitulate otic development in a dish (Koehler et al., 2013). Given the common origin of hair cells and SGNs from otic progenitors and the previously documented appearance of bipolar neurons in the 3D organoid culture, we reasoned it would be possible to actively manipulate cellular fate and induce delamination of otic progenitors and promote their differentiation *in vitro*. The protocol developed by Koehler et al. (2013) efficiently led to hair cell differentiation also in our hands. Moreover, modifying the otic induction step by plating the organoids on 2D Matrigel-coated substrates induced outgrowth of neurons displaying a number of SGN characteristics, including protein/gene expression and electrophysiological features. Gene and protein expression analysis was performed at different time points to support the conclusion that our selected culture conditions guided the cells through the correct stages of otic development. The generated NNE tissue expressing AP2+ and Ecad^+^ was shown to transiently express the placodal markers DLX5 and EYA1 and differentiate into a PAX2/PAX8/Ecad positive otic epithelium. Subsequently, transient upregulation of Neurogenin 1 and NeuroD was detected. We also observed a robust up-regulation of Neurogenin 2 at day 8 of differentiation, despite the lack of *in vivo* evidence for its role in SGN development. Upregulation of GATA3, Prox1, Islet1, p75, Prikle, and MAfb mRNA levels was observed from day 5/8 onward. *In vitro* neuronal delamination of GATA3+, Islet1+, Peripherin+, and Brn3a+ bipolar neurons was then detected by immunostaining analysis.

The expression of GATA3 and Mafb, which we have characterized by qPCR in our neuronal pool, suggests SGN generation at the expenses of vestibular neurons (Appler et al., 2013; Yu et al., 2013). SGNs are never formed in GATA3 conditional deletion mice, whereas VG neurons still do (Duncan and Fritzsch, 2013). Furthermore, higher expression of GATA3 mRNA expression was found in the developing SG compared to the VG (Lu et al., 2011).

The type of neurons derived from truncated protocols (Matrigel only and Matrigel+FGF/LDN) remains unaddressed at the moment. Other sensory neurons, such as trigeminal neurons (Dykes et al., 2010) or dorsal root ganglia (Dykes et al., 2011; Zou et al., 2012) and retinal ganglion cells (Quina et al., 2005) express sensory markers such as Brn3a and intermediate filaments like peripherin. Dissection of the specific lineage would require also in these cases a fine temporal analysis of marker expression. We believe that the neurons obtained in these aborted protocol conditions are not OSNs, given the lack of expression of otic markers at early stages of differentiation (**Figure 2** and **Supplementary Figures S1B,C**).

Spiral ganglion neurons differ along the longitudinal axis of the cochlea for their sensitivity and responsiveness to NT. NT-3 mutations lead to absence of neurons located in the base, while BDNF mutants are more affected in the apical domain (Farinas et al., 2001). In young postnatal animals, the expression of specific ion channels differs along the location in the cochlea. This gives rise to different firing patterns with rapidly adapting, single AP firing neurons located at the base and slowly adapting neurons, firing multiple APs, lying at the apex. Exogenous addition of NT to the culture was shown to modify these firing patterns. Basal neurons were more similar to apical SGNs in presence of NT-3, whereas apical neurons behaving like basal SGNs in presence of BDNF (Adamson et al., 2002a,b; Liu and Davis, 2007; Liu et al., 2014). It is therefore not possible to discriminate between the two types of neurons in our culture, which most probably includes a mixture of the two based on the fact that (1) both NT were provided simultaneously, (2) we observe a mixture of rapid and slow adapting neurons, and (3) the latencies to fire APs can be divided into a faster (10 ms) and slower (39 ms) responding neuronal population, presumptively corresponding to basal and apical turn neurons.

A number of groups have previously attempted to induce human and murine ESC differentiation to SGNs. Combination of FGF2, BMP4, and the NT BDNF and NT3 was used for neuronal differentiation of human ESCs *in vitro* (Shi et al., 2007). A different approach has been established by Chen et al. (2012), guiding human ESCs toward otic progenitors with FGF3 and FGF10, followed by manual colony selection to enrich for neuronal progenitors at the expenses of hair cell progenitors. Alternatively, transient overexpression of Neurogenin-1 has also been used to promote neuronal differentiation from mouse ESCs (Reyes et al., 2008). More recently, an elaborated step-wise protocol to guide cells through NNE, PPE, and later ONP was developed in combination with MACS sorting for enrichment of neuronal progenitors (Matsuoka et al., 2017). In some cases, the derived cells were tested for their functionality by *in vivo* transplantations into the cochlear nerve trunk for SGN replacement (Corrales et al., 2006; Shi et al., 2007; Lang et al., 2008; Reyes et al., 2008; Chen et al., 2012). Re-connection to the sensory epithelium and the cochlear nucleus has been demonstrated histologically in some cases. However, functional recovery has been shown only in one study (Chen et al., 2012). Obviously, the functional reconnection strongly depends on the neuropathy model used, the healthy status of the remaining sensory epithelium, and cochlear nuclei and the secretion of guidance cues that can direct neurons to sprout toward the proper targets. Whether the differences in outcomes with the different transplantation experiments are due to a difference in the “quality” of the generated SGN-like cells or on the hearing loss model used is difficult to dissect. At the same time, a comparison among the different differentiation protocol previously published and ours is also hard to make due to the differences in human versus mouse as well as the lack of direct side-by-side assessment.

## Conclusion

The protocol we present here is based on a step-wise induction of SGN-like cells combining the knowledge acquired in stem cell biology and inner ear development to achieve a rapid, robust, and reproducible induction of sensory otic neurons with the potential to be used in the future for regenerative medicine purposes.

## Author Contributions

MR, MP, C-CT, and SK have performed experiments. MP and MR wrote the manuscript. MR and PS supervised and financed the project.

## Conflict of Interest Statement

The authors declare that the research was conducted in the absence of any commercial or financial relationships that could be construed as a potential conflict of interest.

## Supplementary Material

FIGURE S1 Aberrant differentiation with an incomplete differentiation protocol. **(A)** Schematic of the partial differentiation protocol. **(B)** Heatmap illustration of gene expression profiles after 8 days of differentiation. Fold induction expression compared to undifferentiated ESCs was assessed for the selected genes in two independent experiments undergoing partial/full differentiation. The mean fold induction is shown. Color formatting: blue: low expression, red: high expression. **(C)** Organoids on day 10 of full or partial differentiation protocols immunostained for βIII-tubulin/GATA3, βIII-tubulin/Islet1, βIII-tubulin/Brn3a. Scale bar 100 μm. **(D)** Immunostaining of the derived neurons for βIII-tubulin and Brn3a at day 12. Scale bar 50 μm.
