# Supplementary figures and images for: Generation of Otic Sensory Neurons from Mouse Embryonic Stem Cells in 3D Culture
Source: Front Cell Neurosci. 2017 Dec 19;11:409. doi: 10.3389/fncel.2017.00409 (PMC5742223; doi:10.3389/fncel.2017.00409)

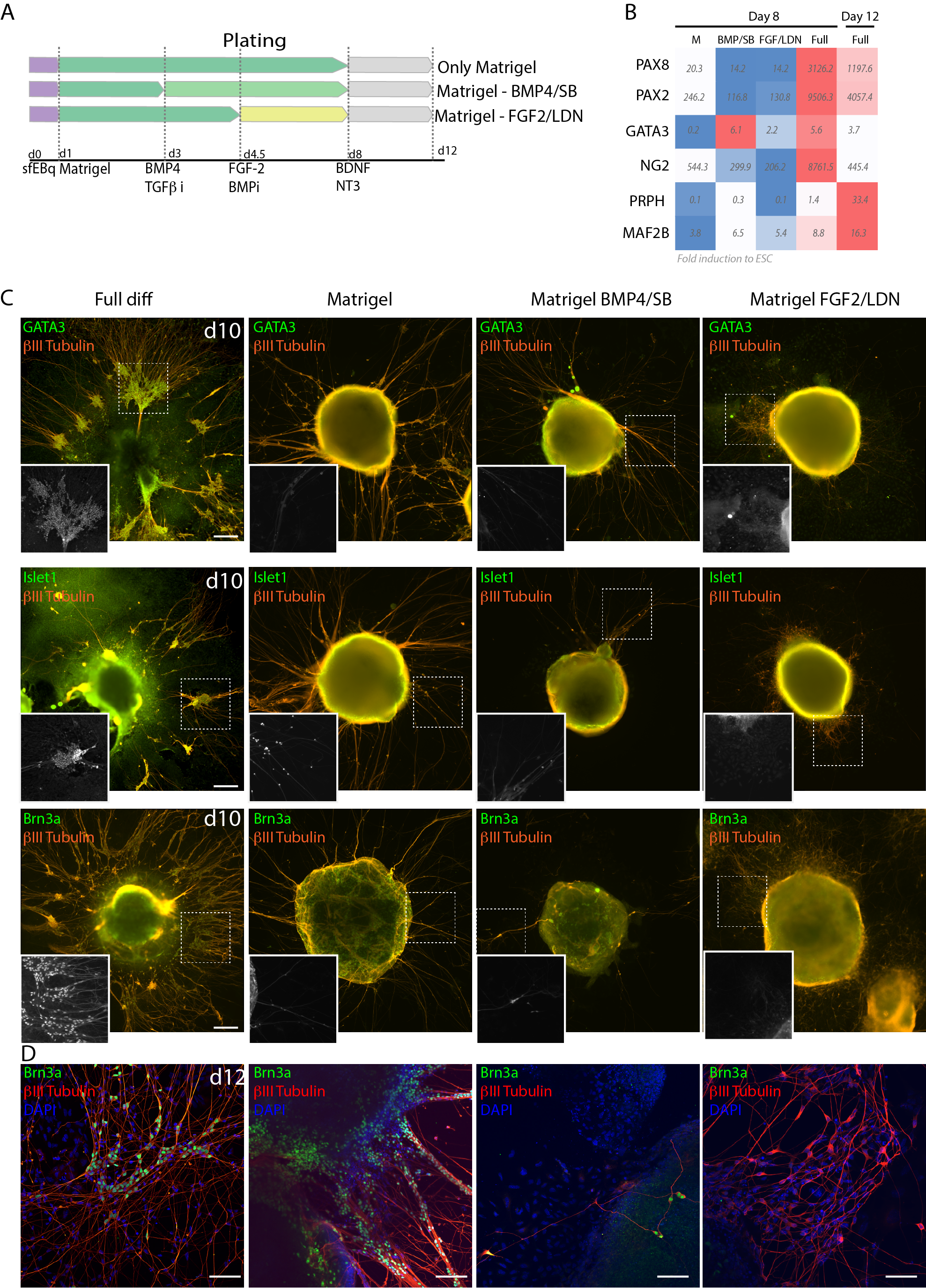

Supplement: FIGURE S1 — Aberrant differentiation with an incomplete differentiation protocol. (A) Schematic of the partial differentiation protocol. (B) Heatmap illustration of gene expression profiles after 8 days of differentiation. Fold induction expression compared to undifferentiated ESCs was assessed for the selected genes in two independent experiments undergoing partial/full differentiation. The mean fold induction is shown. Color formatting: blue: low expression, red: high expression. (C) Organoids on day 10 of full or partial differentiation protocols immunostained for βIII-tubulin/GATA3, βIII-tubulin/Islet1, βIII-tubulin/Brn3a. Scale bar 100 μm. (D) Immunostaining of the derived neurons for βIII-tubulin and Brn3a at day 12. Scale bar 50 μm. [file Image_1.TIF]
